# What Factors Affect Pain Tolerance during Hysteroscopy?

**DOI:** 10.1055/s-0043-1772469

**Published:** 2023-09-08

**Authors:** Cássia Fernanda dos Santos Rosa, Marianna Marques Rodrigues Dourado, Rebecca Schuster Dorea Leite, Laís Viana Aragão Almeida, Brenda Lima Meireles Martins, Johnnatas Mikael Lopes

**Affiliations:** 1 Universidade Tiradentes, Aracaju, SE, Brazil; 2 Universidade Federal do Vale São Francisco, Paulo Afonso, BA, Brazil

Hysteroscopy is considered the gold standard for the evaluation and management of intrauterine pathologies because it is capable of simultaneously offering diagnosis and treatment for many of them. Genital tract infections, pregnancy, pelvic inflammatory disease, active herpetic infections, or human papilloma virus infections are contraindications for its performance. The indications are diverse, including suspected intracavitary mass, abnormal endometrial thickening, infertility, congenital anomalies, intrauterine adherence, in addition to post-treatment follow-up, and biopsy may be performed when necessary. 1

Initially, outpatient hysteroscopy was restricted to diagnostic procedures. With advances in technology, there has been an increase in their practice in the office, rather than in the operating room with anesthesia. Despite this, the outpatient method is associated with higher levels of pre- and intraprocedure anxiety, which impairs patient satisfaction with the intervention and is associated with a greater perception of pain. 2 Furthermore, higher levels of pain during the intervention were associated with high rates of refusal to perform the procedure in the future, as well as higher rates of unsuccessful procedures. 3 Thus, the impact of pain on the continuity of health care by patients is notorious, as well as its interference in the adequate control of their pathologies.

The article by Coimbra et al., 4 entitled Predictive Factors of Tolerance in Office Hysteroscopy – a 3-Year Analysis from a Tertiary Center, addresses a topic of great relevance in the management of women's health. However, we would like to point out suggestions for improvement in the production of results that are of practical use.

We understand that in longitudinal and cross-sectional research designs, where the investigated outcome has a high prevalence or incidence (> 10%), the effect measure used to estimate the relationship/prediction between the independent variable and the outcome cannot be the odds ratio estimated by logistic regression. 5 Applying this analytical approach produces overestimated point and interval estimates. The best strategy is the application of Poisson or Cox regression, depending on the type of outcome, generating measures such as relative risk or hazard ratio, respectively. 5

In the study by Coimbra et al., 4 it is possible that the oversizing interfered with the significance of the age variable and the risk values of the other variables presented in Table 2, since the considered outcome is low tolerance (terrible and poor), which has an incidence of 14.9%. In addition, the reason for not including the variable intracavitary pathology, which revealed a difference in the outcome, and others such as parity number, was not identified in the model.

It is extremely important to create a raw model with the estimates of effects for the independent variables as a whole and the selection criteria for them to appear in the final model. Commonly, factors may have a marginal relationship with the outcome in the crude analysis and in the adjusted model if it reveals a significant predictor, such as age, for example. Furthermore, overestimated estimates may limit findings in systematic reviews with meta-analysis.
